# Health Care Cost Analyses for Exploring Cost Savings Opportunities in Older Patients: Longitudinal Retrospective Study

**DOI:** 10.2196/10254

**Published:** 2018-08-01

**Authors:** Stephen Agboola, Mariana Simons, Sara Golas, Jorn op den Buijs, Jennifer Felsted, Nils Fischer, Linda Schertzer, Allison Orenstein, Kamal Jethwani, Joseph Kvedar

**Affiliations:** ^1^ Connected Health Innovation Partners Healthcare Boston, MA United States; ^2^ Department of Chronic Disease Management Philips Research Eindhoven Netherlands; ^3^ Philips Lifeline Framingham, MA United States

**Keywords:** health care cost analysis, cost acuity pyramid, patient segmentation, multicenter study, Markov chains

## Abstract

**Background:**

Half of Medicare reimbursement goes toward caring for the top 5% of the most expensive patients. However, little is known about these patients prior to reaching the top or how their costs change annually. To address these gaps, we analyzed patient flow and associated health care cost trends over 5 years.

**Objective:**

To evaluate the cost of health care utilization in older patients by analyzing changes in their long-term expenditures.

**Methods:**

This was a retrospective, longitudinal, multicenter study to evaluate health care costs of 2643 older patients from 2011 to 2015. All patients had at least one episode of home health care during the study period and used a personal emergency response service (PERS) at home for any length of time during the observation period. We segmented all patients into top (5%), middle (6%-50%), and bottom (51%-100%) segments by their annual expenditures and built cost pyramids based thereon. The longitudinal health care expenditure trends of the complete study population and each segment were assessed by linear regression models. Patient flows throughout the segments of the cost acuity pyramids from year to year were modeled by Markov chains.

**Results:**

Total health care costs of the study population nearly doubled from US $17.7M in 2011 to US $33.0M in 2015 with an expected annual cost increase of US $3.6M (*P*=.003). This growth was primarily driven by a significantly higher cost increases in the middle segment (US $2.3M, *P*=.003). The expected annual cost increases in the top and bottom segments were US $1.2M (*P*=.008) and US $0.1M (*P*=.004), respectively. Patient and cost flow analyses showed that 18% of patients moved up the cost acuity pyramid yearly, and their costs increased by 672%. This was in contrast to 22% of patients that moved down with a cost decrease of 86%. The remaining 60% of patients stayed in the same segment from year to year, though their costs also increased by 18%.

**Conclusions:**

Although many health care organizations target intensive and costly interventions to their most expensive patients, this analysis unveiled potential cost savings opportunities by managing the patients in the lower cost segments that are at risk of moving up the cost acuity pyramid. To achieve this, data analytics integrating longitudinal data from electronic health records and home monitoring devices may help health care organizations optimize resources by enabling clinicians to proactively manage patients in their home or community environments beyond institutional settings and 30- and 60-day telehealth services.

## Introduction

The United States spends more on health care per person than any other country in the world [1]. National health care expenditures increased by 5.8% to US $3.2 trillion from 2014 to 2015, or US $9990 per person, and accounted for 17.8% of gross domestic product [2]. A recent study [3] on 5 fundamental factors associated with increases in US health care spending, including population size, population age structure, disease prevalence or incidence, service utilization, and service price and intensity, found that increases in service price and intensity were associated with a 50% health care spending increase. Increases in population size and age were also positively associated with increased health care spending, whereas changes in disease prevalence or incidence were negatively associated.

A sizable proportion (20%) of all national health care expenditures are due to Medicare spending, a federal health insurance program for US citizens who are 65 years and older, younger people with certain disabilities, and those who suffer from end stage renal disease [4]. For each consecutive year from 2011 to 2015, national average Medicare expenditures per enrollee steadily increased from US $11,408, US $11,465, US $11,509, US $11,711, to US $11,951 [5]. Factors contributing to this growth included rising medical costs and an expansion of health insurance from 2014 to 2015, which increased the use of health services [5]. Among Medicare beneficiaries, older patients are among the groups that spend the most, and this is driven largely by inpatient (including emergency care) and postacute care costs [6]. In fact, in 2015, 35.9 per 100 individuals between the ages of 65 to 74 years had an emergency visit compared with 60.5 per 100 individuals aged 75 years and older [7]. Medicare beneficiaries are nearly twice as likely as the privately insured to be admitted 4 or more times per year to the emergency department (ED) [8]. Further, readmissions are common among Medicare patients and cost US $26 billion annually, as estimated by the Agency for Healthcare Research and Quality [9]. Nearly a quarter of these Medicare readmissions are considered potentially avoidable [10,11] by the Centers for Medicare & Medicaid Services (CMS), the federal agency that administers Medicare.

Unsustainable health care costs and the need to improve overall efficiency is the driving force for the introduction of value-based care, wherein clinicians aim to cost effectively monitor, diagnose, and treat patients. Many health care organizations (HCOs) now use value-based care strategies [12], such as connected solutions that seamlessly integrate sources of big data and data analytics to identify and manage high-risk and high-cost patients [13]. An example of technology that is used worldwide for older patients is the personal emergency response service (PERS). PERS is designed to promote independent living in older adults by providing help in case of medical emergencies that could lead to costly ED visits and hospitalizations. Although PERS has been widely used for many years to monitor older patients, only recently has PERS data been utilized to develop CareSage [14], a data analytics engine that utilizes PERS device data to identify older patients at risk of ED transports/visits. Further, the unique combination of electronic health records (EHRs) and PERS data improved the existing ED transports predictive model and facilitated the development of new models predicting emergency care [15]. However, to enable the development of cost-effective population health programs for older patients utilizing PERS, there is a need to better understand their health care utilization costs.

Health care expenditures in the United States are unevenly distributed across individuals and different segments of the population [16-20]. For example, the bottom 50% of the population (B segment which includes the 50% less expensive patients) spends only 3%-4% on health care, whereas the top 5% of the population (T segment, which includes the 5% most expensive patients) spends 50% of the total expenditures. The middle 45% of the population (M segment) accounts for the remaining 45% of the total cost. Accordingly, most HCOs focus on developing population health management programs targeting the most expensive patients in the T segment. The persistence in the health care cost of the T segment has been explored in a few studies that justify the use of targeted interventions [21-24]. However, none of these studies have examined the nonpersistence of health care costs, (ie, the full dynamics of patient and cost flows between the different segments from year to year). Furthermore, little is known about patient and cost flow prior to reaching the top 5%. To address these gaps and enable HCOs to deliver targeted and cost-effective interventions, we analyzed patient flow throughout the cost segments and associated annual health care cost changes.

## Methods

### Aims

The primary aim of this study was to evaluate the health care costs of older patients using PERS over a period of 5 years. Specifically, to answer the following questions:

- What is the total health care cost of the study population from fiscal year 2011 to fiscal year 2015 (FY11-FY15) and its distribution across specific cost segments?
- Are there longitudinal trends in health care cost across the cost segments?
- How many patients are moving up/down the cost segments and how do their health care costs vary annually?

### Design

This was a retrospective, longitudinal, multicenter study to evaluate health care costs of inpatient and outpatient hospital encounters in patients using PERS for any length of time during the study period of 5 years (FY11-FY15). The study was conducted using US data and was approved by the Partners Human Research Committee, the Institutional Review Board for Partners Healthcare hospitals.

### Settings

Study participants were identified from Partners Healthcare at Home (PHH), a home health agency that offers general care as well as specialized services to help patients within the Partners Healthcare System (PHS) network of hospitals to manage chronic conditions while at home. Patients are usually referred to the PHH service by their care providers after discharge from the hospital. In addition to in-person home visits, PHH utilizes a variety of health care technologies to manage their patients. One of these technologies is the Lifeline PERS, which PHH care providers routinely recommend to chronically ill patients who are at risk of falls or other health-related emergencies. Detailed descriptions of PHH and PERS were described in a previous paper [25].

### Subject Selection

**Figure 1:**
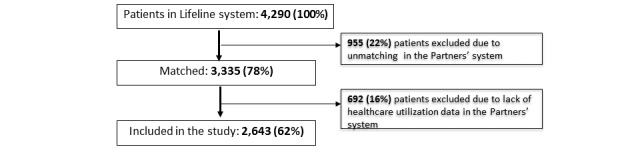
Flow chart diagram of the study subjects.

Subjects included in this study received health care at any of the 5 PHS affiliated hospitals and had at least one inpatient and/or outpatient encounter. Study subjects had at least one episode of PHH care with average duration of 2-3 months and were enrolled to PERS through PHH for any length of time during FY11-FY15. Initially, there were 4290 patients identified as PERS users from the Lifeline database, as illustrated in Figure 1. We excluded patients that were unmatched (by first name, last name, and date of birth) in the PHS data warehouse and those without any health care utilization record in the study period because their health care costs were zero without any variation. This resulted in 2643 patients included in the data analysis. All data were deidentified before analyses.

### Data Sources

The primary data source for this study was the enterprise data warehouse (EDW), an electronic medical record data repository for hospitals within the PHS network. It includes data such as patient demographics, medical conditions, clinical encounters, and health care costs. Health care cost data in EDW is obtained from the PHS costing system (ie, billing and internal cost to the hospital); it does not refer to insurer payment or cost to the patient. “Total cost” is the sum of variable and fixed costs for direct and indirect patient care during hospital inpatient and outpatient encounters. Hospital costing data are divided into fiscal years (FYs), as opposed to calendar years, with the fiscal year beginning Oct 1, (eg, FY11 begins on 2010 Oct 1). All mention of “year” herein refers to the fiscal year.

The PERS database included patient demographics, living situation, caregiver network, self-reported medical conditions, and medical alert data. The latter included all information gathered during the interactions of the patients with Lifeline call center associates when the PERS help button was pressed, including the reasons for pressing and the outcomes of the interactions.

### Subject Segmentation

**Figure 2:**
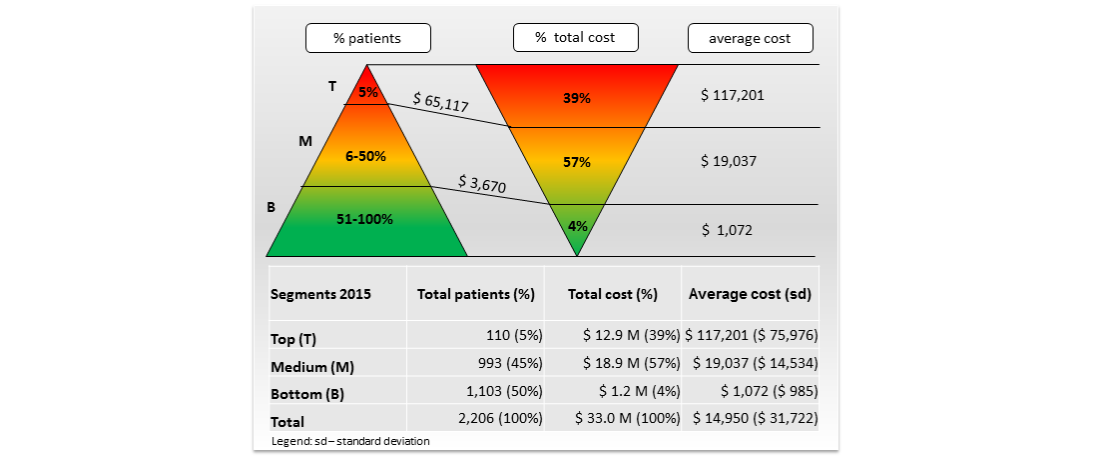
Cost acuity pyramid based on health care cost in 2015.

The subject segmentation was based on the following steps performed for each fiscal year (FY11-FY15). Firstly, we selected the patients that had any health care costs in a particular FY from all 2643 patients included in the study. Secondly, we calculated the annual cost of each patient as the sum of the total costs of their inpatient and outpatient encounters. Third, we ranked subjects by their annual health care costs from highest to lowest. Finally, we grouped them into the following segments: T segment constitutes the top 5% (0%-5%) most expensive patients; M segment comprises the middle 45% (5%-50%) of all patients; B segment includes the bottom 50% (50%-100%) least expensive patients. We visualized these 3 segments for each fiscal year by an annual cost acuity pyramid, as illustrated in Figure 2. The cost acuity pyramid is a core visual in this paper and is instrumental in illustrating the disproportion between the size of the segments and their health care costs.

### Outcomes

To address the aforementioned study objectives, our primary outcomes were to quantify patients who moved up, down, or stayed in the same segment of cost acuity pyramids over a 2-year period and to evaluate the costs associated with these flows.

Prior to analyzing the primary outcomes, we conducted exploratory analyses to evaluate a secondary outcome of the total health care cost of the study population and its distribution across the segments of the cost acuity pyramids for each available fiscal year. In addition, we performed inferential analysis to identify longitudinal trends in the total health care costs of the complete study population and each segment of the cost acuity pyramid.

### Statistical Analysis

Demographic and health care utilization data for FY11-FY15 were extracted from EDW using Microsoft Structured Query Language Server Management Studio (SSMS) 2014. Data management and deidentification were achieved through SSMS and Microsoft Excel 2007. The statistical analysis described below was performed via R version 3.4.1 [26].

To evaluate our primary outcomes, we applied a 3-step analysis, which included the following steps: model the patients’ flow between the T, M, and B segments of the cost acuity pyramid over each 2-year period, group these flows to quantify patients moving up, down, or staying at the same segment of the cost acuity pyramid, and estimate the cost flow associated with the patient flow.

**Figure 3:**
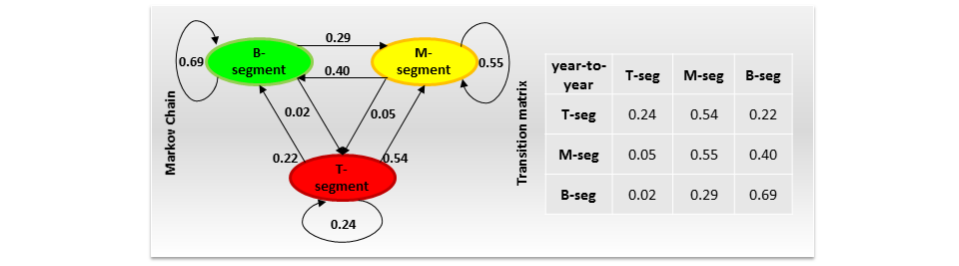
Markov chain of the patient flow and associated transition matrix.

To model the patients’ flows in step 1 above, we created a Markov chain of the flow from each segment to all others over 2 successive FYs. A Markov chain describes a sequence of possible events, in which the probability of each event depends only on the state attained in the previous event. Markov chains have been used in the economic evaluation of health care [27] but not to examine the flow between the T, M, and B segments of the cost acuity pyramid. The Markov chain we built in this study included 3 states (B, M, or T segment) and 9 transitions (B to B, B to M, B to T, M to B, M to M, M to T, T to B, T to M, and T to T). These transitions could be grouped into 3 persistent (B to B, M to M, and T to T) and the remaining 6 nonpersistent transitions. The states represent the patient segments of the cost acuity pyramid, whereas the transitions indicate the probability that a patient will move from one segment to another over a 2-year period. The probability of transition change is an average of the flow percentages over 4 sequential pairs of FYs, that is, FY11-FY12, FY12-FY13, FY13-FY14, and FY14-FY15. The 9 probabilities constitute a 3×3 transition matrix associated with the Markov chain, as illustrated in Figure 3. This transition matrix was used in step 2 of the analysis described above. Namely, the probabilities in the lower triangular, upper triangular, and diagonal of this matrix were multiplied by the size of the corresponding segments and summed to quantify patient movements throughout the segments of the cost acuity pyramid.

To evaluate health care expenditure trends, we conducted linear regression analyses. Four linear regression models were built with health care costs of the total study population and T, M, and B segments as the dependent variables with each available fiscal year serving as the independent variable. Each model provided an estimate of the expected annual cost increases/decreases.

## Results

### Characteristics of Study Population

The study population was, on average, 79 years old, predominately female (1990/2643, 75.29%), white (2312/2643, 93.41%), living alone (2483/2643, 93.95%), without family caregivers (2629/2643, 99.47%), and at least 86.70% (1310/1511) had a high school education (Table 1). The majority of the patients (1728/2643, 65.38%) had multiple medical conditions.

### Health Care Cost Distribution and Trends

Health care costs were unevenly distributed across the segments of the cost acuity pyramid for each fiscal year. For example, there were 2206 patients with any health care utilization in 2015, as illustrated in Figure 2. Their total health care costs were US $33.0M and the average cost per patient was US $14,950 (SD US $31,722). The T segment in 2015 (in total 110 patients with annual cost above US $65,117) constituted 39% (US $12.9M) of the total health care expenditures and the average cost per patient was US $117,201 (SD US $75,976). The M segment (in total 993 patients with annual cost above US $3670) accounted for 57% (US $18.9M) of the total health care expenditures and the average cost per patient was US $19,037 (SD US $14,534). The B segment (in total 1103 patients) comprised only 4% (US $1.2M) of the total health care expenditures and the average cost per patient was US $1072 (SD US $985).

**Figure 4:**
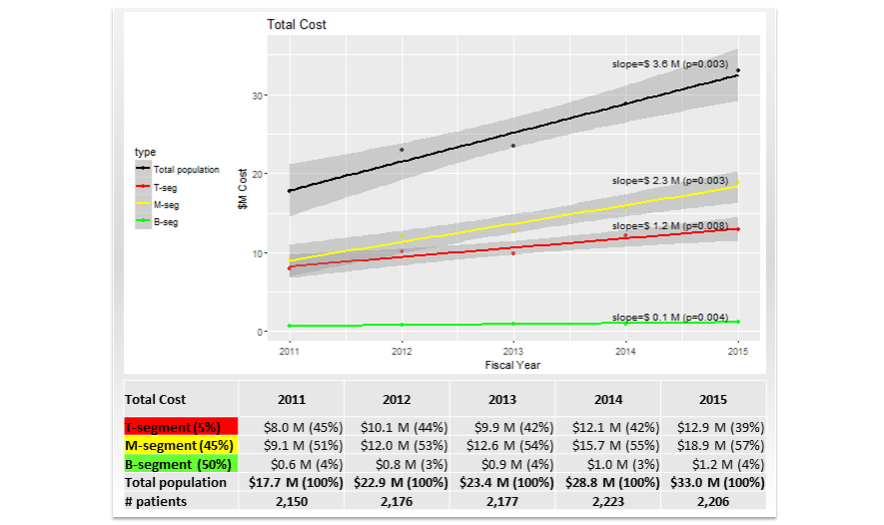
Health care cost trends of total population, top (T), middle (M), and bottom (B) segments from 2011 to 2015.

The total health care expenditure of the study population nearly doubled from US $17.7M in FY11 to US $33.0M in FY15, although the number of patients per year having any costs remained similar, as illustrated in Figure 4. About two thirds of the total expenditure (ranging from 63% to 71% throughout FY11-FY15) included hospital admissions costs, which doubled from US $11.4M in FY11 to US $23.4M in FY15. The remaining one third of the total expenditure was outpatient encounters costs, which also increased from US $6.3M in FY11 to US $9.6M in FY15.

The M segment was the most expensive with total costs increasing from US $9.1M in FY11 to US $18.9M in FY15, as illustrated in Figure 4. Moreover, the relative contribution of the M segment to the total cost increased from 51% in FY11 to 57% in FY15. Next was the T segment, the costs of which increased from US $8.0M in FY11 to US $12.9M in FY15. The relative contribution of the T segment to the total cost decreased from 45% in FY11 to 39% in FY15, and this was in contrast to the M segment. The cost of the B segment increased from US $0.6M in FY11 to US $1.2M in FY15. However, the relative contribution of the B segment to the total cost remained steady at 3%-4% over the 5 years. Further, linear regression analysis showed that the increasing trend in total health care costs of the study population was statistically significant (*P*=.003) with an expected annual cost increase of US $3.6M, as illustrated in Figure 4. This growth was driven primarily by the significant cost increase of US $2.3M in the M segment (*P*=.003). The expected annual cost also increased significantly in the T and B segments with US $1.2M (*P*=.008) and US $0.1M (*P*=.004), respectively. The trends in both components of total cost, inpatient and outpatient costs, were similar to those illustrated in Figure 4.

### Patients and Cost Flow Throughout Segments of the Cost Pyramids

The Markov model of the patient flow throughout the segments of the cost acuity pyramid is illustrated in Figure 3.

**Figure 5:**
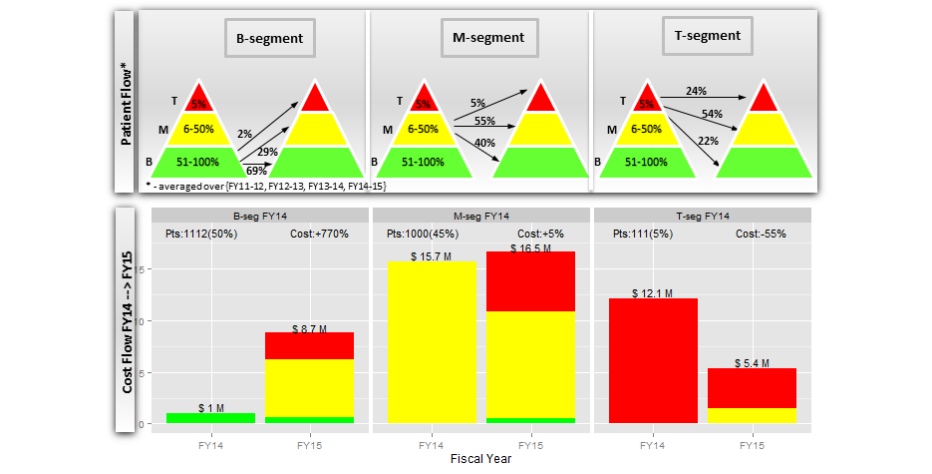
Patient and cost flows of top (T), middle (M), and bottom (B) segments of cost acuity pyramid.

An alternative visualization using the cost acuity pyramids is shown in the upper part of Figure 5. Both figures highlight several important insights. First, the B segment was the most stable of all 3 segments. A majority (69%) of the patients in the B segment stayed in the same segment during the next fiscal year, 2% moved up to the T segment, and the remaining 29% of the patients moved up to the M segment of the cost acuity pyramid during the next fiscal year. Second, the M segment was more dynamic than the B segment. A majority (55%) of the patients in the M segment stayed in the same segment during the next fiscal year, 5% moved up to the T segment, and the remaining 40% moved down to the B segment of the cost acuity pyramid in next fiscal year. Third, the T segment was the most dynamic of all 3 segments. Only 24% of the patients in the T segment stayed in the same segment next fiscal year, whereas 54% and 22% of the patients moved down to the M and B segments of the cost acuity pyramid during the next fiscal year, respectively.

The cost flow associated with the patient flow is depicted in the lower part of Figure 5, specifically for the two most recent FYs, FY14 and FY15. The cost of 1112 patients in the B segment increased from US $1M in FY14 to US $8.7M in FY15 (+770%) owing to their movement up to the M and T segments, as depicted in the upper part of Figure 5. Similarly, the cost of 1000 patients in the M segment increased from US $15.7M in FY14 to US $16.5M in FY15 (+5%) owing to their movements to the B and T segments. The cost of 111 patients in the T segment decreased from US $12.1M in FY14 to US $5.4M in FY15 (−55%) owing to their movement down to the lower segments.

We evaluated the potential demographic differences between patients who moved up, stayed, or moved down the cost acuity pyramid, as detailed in Table 2. Using the patient flow from FY14 to FY15 as an example, the patient groups were statistically similar to each other, except for likelihood of living alone and the number of comorbid medical conditions. More specifically, patients who stayed in the same segment were most likely to live alone and those who moved down being least likely to live alone (*P*=.02). Further, patients who stayed in the same segment were higher in proportion with none of the selected comorbidities compared with patients who moved up or down (*P*<.01). Patients who moved up were more likely to have 4 or more of the selected comorbid conditions (*P*=.03) than those who stayed or moved down.

**Figure 6:**
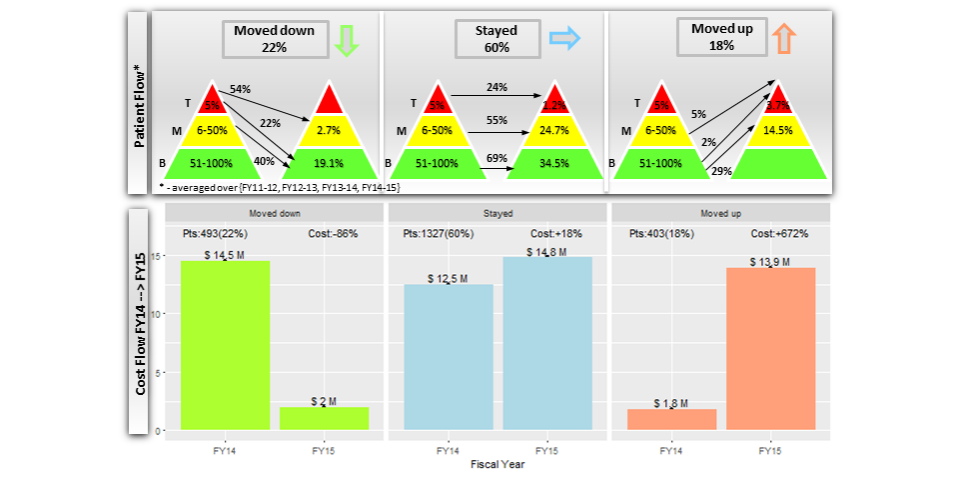
Patients moving throughout the cost acuity pyramid and associated cost flow.

After quantifying the patient and cost flows throughout the segments of the cost acuity pyramids, we evaluated the primary outcome of how many patients moved up, down, or stayed in the same segment the following year, as illustrated in Figure 6. In total, 22.18% (493/2223) of the patients moved at least one segment down the cost acuity pyramid yearly and their costs decreased from US $14.5M in FY14 to US $2.0M in FY15 (-86%). Another 18.13% (403/2223) of the patients moved at least one segment up the cost acuity pyramid yearly and their costs increased from US $1.8M in FY14 to US $13.9M in FY15 (+672%). Overall, 59.70% (1327/2223) of patients stayed in the same segment of the cost acuity pyramid yearly; however, their costs also increased from US $12.5M in FY14 to US $14.8M in FY15 (+18%).

**Table 1:** Characteristics of the study population.

Variables		Study population (N=2643), mean (SD) or n (%)^a^
Age, mean (SD)		79 (11)
**Age category, n (%)**		
	<65	303 (11.46)
	65+	2340 (88.54)
**Gender, n (%)**		
	Female	1990 (75.29)
**Race (N=2475)^b^, n (%)**		
	White	2312 (93.41)
	Hispanic	9 (0.36)
	Black/African American	128 (5.17)
	Other	26 (1.05)
**Family caregivers, n (** **%)**		
	0	2629 (99.47)
	1	14 (0.53)
**Live alone, n (%)**		
	Yes	2483 (93.95)
	No	160 (6.05)
**Education (N=1511)^c^, n (%)**		
	≥College	551 (36.47)
	Some college	102 (6.75)
	High school	657 (43.48)
	<High school	201 (13.30)
**Marital status (N=2374)^d^, n (%)**		
	Married	695 (29.28)
	Divorced	317 (13.35)
	Single	475 (20.00)
	Widowed	887 (37.36)
**Medical condition, n (%)^e^**		
	0	386 (14.60)
	1	529 (20.02)
	2	562 (21.26)
	3	473 (17.90)
	≥4	693 (26.22)

^a^Percentages may not add to 100 due to rounding.

^b^Unknown: n=168.

^c^Unknown: n=1132.

^d^Unknown: n=269.

^e^Selected medical conditions included disordered lipid metabolism, atrial fibrillation, congestive heart failure, chronic obstructive pulmonary disease, malignant cancer, fractures, pneumonia, obesity, and acute myocardial infarction.

**Table 2:** Characteristics of study population (N=2223) who moved up, stayed, or moved down the cost acuity pyramid from fiscal year 2014 to fiscal year 2015.

Variables		Moved up^a^		Stayed^a^		Moved down^a^		*P* value	
Total, N (%)		403 (18)		1327 (60)		493 (22)			
Age, mean (SD)			10.9 (78.9)		11 (78.8)		11 (78.6)		.91
**Age category, n (%)**								.96	
	<65		48 (11.91)		162 (12.21)		58 (11.76)		
	65+		355 (88.09)		1165 (87.79)		435 (88.24)		
**Gender, n (%)**								.73	
	Female		306 (75.93)		1000 (75.36)		364 (73.83)		
**Race, n (%)^b^**		**N=391**		**N=1259**		**N=468**		.86	
	White		368 (94.12)		1169 (92.85)		433 (92.52)		
	Hispanic		1 (0.26)		6 (0.48)		1 (0.21)		
	Black/African American		17 (4.35)		71 (5.64)		30 (6.41)		
	Other		5 (1.28)		13 (1.03)		4 (0.85)		
**Family caregivers, n (%)**								.42	
	None		402 (99.75)		1319 (99.40)		492 (99.80)		
**Live alone, n (%)**								.02	
	Yes		375 (93)		1255 (95)		449 (91)		
**Education, n (%)^c^**		**N=255**		**N=758**		**N=295**		.86	
	≥College		98 (38.43)		282 (37.20)		104 (35.25)		
	Some college		19 (7.45)		38 (5.01)		21 (7.12)		
	High school		103 (40.39)		330 (43.54)		126 (42.71)		
	<High school		35 (13.73)		108 (14.25)		44 (14.92)		
**Marital status, n (%)^d^**		**N=366**		**N=1200**		**N=444**		.94	
	Married		109 (29.78)		352 (29.33)		126 (28.38)		
	Divorced		49 (13.39)		166 (13.83)		65 (14.64)		
	Single		64 (17.49)		237 (19.75)		90 (20.27)		
	Widowed		144 (39.34)		445 (37.08)		163 (36.71)		
**Medical condition, n (%)^e^**								<.01	
	0		16 (3.97)		149 (11.23)		31 (6.29)		<.01
	1		55 (13.65)		249 (18.76)		91 (18.46)		.06
	2		94 (23.33)		297 (22.38)		112 (22.72)		.92
	3		92 (22.83)		245 (18.46)		109 (22.11)		.07
	≥4		146 (36.23)		387 (29.16)		150 (30.43)		.03

^a^Percentages may not add to 100 due to rounding.

^b^Unknowns: moved up: n=12; stayed: n=68; moved down: n=25.

^c^Unknowns: moved up: n=148; stayed: n=569; moved down: n=198.

^d^Unknowns: moved up: n=37; stayed: n=127; moved down: n=49.

^e^Selected medical conditions included disordered lipid metabolism, atrial fibrillation, congestive heart failure, chronic obstructive pulmonary disease, malignant cancer, fractures, pneumonia, obesity, and acute myocardial infarction.

## Discussion

### Principal Findings

This study is the first to quantify patients’ annual movements through the segments of the cost acuity pyramid and associated changes in health care costs. We discovered 3 main findings. First, the total health care cost of the study population doubled from US $17.7M to US $33.0M (FY11-FY15) with an expected annual increase of US $3.6M (*P*=.003). Second, patients in the M segment were major contributors to the increased cost with an expected annual increase of US $2.3M (*P*=.003). The M segment was consistently the costliest throughout all 5 FYs. Third, the patient and cost flow analysis showed that 18% of patients moved up the cost acuity pyramid yearly and their costs increased by 672%. In contrast, 22% of patients moved down with cost reductions of 86%. Although the remaining 60% of patients stayed in the same segment from year to year, their health care costs also increased by 18%.

Our first finding is consistent with those of the prior studies characterizing high-cost users as predominantly older patients with functional limitations and multiple chronic conditions [28-30], yet the magnitude of this annual increase of 20% (US $3.6 M) was notably 3 times higher than the national average of 6% per year projected by CMS [31]. This discrepancy can be explained, in part, by the fact that our population was significantly older than the national CMS population (79 vs 71 years old) [25]. Nevertheless, we found in a previous study [25] that 37% of all costly admissions were due to medical conditions leading to potentially avoidable admissions [10,11]. Taken together, this suggests that interventions targeting these conditions may be an effective strategy for in older adults.

The second finding that the M segment (not the T segment) of the cost acuity pyramid was the most expensive each year is a new insight that reveals the importance of the M segment for cost management. Currently, most HCOs develop population health management programs targeting the T segment of the cost acuity pyramid [32-34]. Although these programs have demonstrated improvement in clinical outcomes, evidence supporting their impact on health care costs is inconclusive [35]. Often, these studies compare health care expenditures pre- and postprogram introduction. The lack of randomized control trials raises the question of whether the reported cost savings can be attributed to the effect of the interventions or a statistical phenomenon known as regression to the mean [36]. Figure 5 supports the latter, illustrating that a majority (76%) of patients in the T segment tended to move to the M and B segments the following fiscal year and consequently, their costs dropped by US $6.7M (−55%). However, this cost reduction can be completely phased out by the US $7.7M (770%) cost increase of the B segment owing to patients moving up. Therefore, for all cost reduction initiatives, the unforeseen costs of patients moving up the cost acuity pyramid, which are hidden within an overall budget, may seem to invalidate the work being done to manage the costs of the T segment.

The third finding illustrates how health care expenditures of the different segments of the cost acuity pyramid changed over the 2-year period. Previous work [25] analyzing the persistence of expenditures over a 2-year period reported a slightly higher percentage of patients remaining in the T segment (34% vs 24%) and B segments (73% vs 69%) than that reported by us. However, this study involved the general US population, which is much younger than our study population. Our study is the first one that quantifies not only the patients staying at the same cost segment (persistent flows) but also those moving up and down throughout the segments of the cost acuity pyramid (nonpersistent flows) over the sequential 2-year period as well as their cost changes. Analyzing cost persistence over 3-, 4-, or 5-year periods is more appealing than over a 2-year period. However, our choice to analyze over a 2-year period was imposed by the growing complexity of the nonpersistent flows. Each subsequent year the number of nonpersistent flows tripled from 6 to 18 to 54 to 162 over 2-, 3-, 4-, and 5-year periods, respectively, whereas the number of persistent flows stayed the same at 3. Further, analyzing over a 2-year period kept the number of patients in the nonpersistent flows still meaningful for statistical analyses.

In evaluating potential group differences in patients who moved up, stayed, or moved down the cost acuity pyramids, we observed that patients who stayed in the same segment were more likely to live alone and to have fewer comorbid conditions. Patients who moved up the cost acuity pyramid had the highest proportion of comorbid conditions. Future work will examine additional patient characteristics.

In summary, our findings demonstrate that a holistic cost management approach is needed to attenuate the overall increases in total health care costs, taking into account the dynamic flows between all segments of the cost acuity pyramid, rather than the T segment only. This approach would target interventions to patients at risk of moving up the cost acuity pyramids.

### Limitations

This study had a number of limitations. Firstly, PERS used by this population was self-paid and may limit the study generalizability to patients that could afford the service. Secondly, our analyses did not include the costs of patients’ clinical encounters that may have occurred outside the Partners Health care network. Further, information about patients’ alignment with insurers accepted by PHS at the time of their health care utilization was not available because the dataset was derived from EHR, rather than the insurance claims. Thirdly, other types of health care costs, such as skilled nursing facilities and home health agencies, are not included in our analysis because of data unavailability. Finally, this analysis was conducted using US data from the PHH population; therefore, other population results may vary.

### Future Studies

Future work will investigate which patient characteristics have the potential to predict patient flow from year to year, including hospital utilization, encounter-level principal diagnoses and procedures, in addition to the patient demographics evaluated herein. We will also evaluate whether these characteristics are static or dynamic over time. Additionally, we will conduct a prospective study to evaluate the cost savings of disease management programs for older patients using PERS and CareSage as a long-term home monitoring service [37].

### Conclusions

Although many HCOs target intensive and costly interventions to their most expensive patients, this analysis unveiled potential cost savings opportunities by managing the patients in the lower cost segments that are at risk of moving up the cost acuity pyramid. Accordingly, HCOs should prioritize population health management programs able to identify patients at risk of moving up the cost acuity pyramid and provide interventions tailored to a patient’s specific problem, which might be related to frequent ED transports/visits, medication nonadherence, or lack of social support. To achieve this, data analytics integrating longitudinal data from the EHRs and home monitoring devices may help HCOs optimize resources by enabling clinicians to proactively manage patients in their home or community environments, beyond institutional settings, and in 30- or 60-day telehealth services.

## Abbreviations

CMS: Centers for Medicaid and Medicare Services
ED: emergency department
EDW: enterprise data warehouse
EHR: electronic health record
FY: fiscal year
HCO: health care organizations
PERS: personal emergency response service
PHH: Partners Healthcare at Home
PHS: Partners Healthcare System
SSMS: structured query language server management studio
